# Draft Genome Sequence of *Fusobacterium necrophorum* subsp. *funduliforme* Bovine Liver Abscess Isolate B35

**DOI:** 10.1128/genomeA.00412-14

**Published:** 2014-05-01

**Authors:** Michael J. Calcutt, Mark F. Foecking, Tiruvoor G. Nagaraja, George C. Stewart

**Affiliations:** a Department of Veterinary Pathobiology, University of Missouri, Columbia, Missouri, USA; b Department of Diagnostic Medicine/Pathobiology, Kansas State University, Manhattan, Kansas, USA; c Bond Life Science Center, University of Missouri, Columbia, Missouri, USA

## Abstract

*Fusobacterium necrophorum* is a Gram-negative anaerobic bacterium that causes foot rot and liver abscesses in cattle. *F. necrophorum* subsp. *necrophorum* and the less virulent organism *F. necrophorum* subsp. *funduliforme* are recognized. We present here a draft genome sequence of the bovine liver abscess isolate *F. necrophorum* subsp. *funduliforme* strain B35, which affords a genomic perspective of virulence and bovine adaptation.

## GENOME ANNOUNCEMENT

*Fusobacterium necrophorum* is a normal inhabitant of the bovine rumen but is also associated with bovine disease (1). *F. necrophorum* subsp. *necrophorum* (formerly *F. necrophorum* biotype A) is the principal pathogen of cattle (causing liver abscesses, foot rot, and calf diphtheria), whereas *F. necrophorum* subsp. *funduliforme* (biotype B), when isolated from liver abscesses, is more commonly recovered in mixed culture. Accordingly, *F. necrophorum* subsp. *funduliforme* is generally considered to be less virulent in the bovine host (2). The subspecies differ in a number of characteristics (1), but these have not been delineated at the genomic level. Important to human health, *F. necrophorum* subsp. *funduliforme*-like organisms are also associated with Lemierre’s syndrome, a syndrome characterized by suppurative thrombophlebitis of the internal jugular vein, bacteremia, and metastatic abscesses (3, 4). Draft genome sequences have been determined for four human origin *F. necrophorum* subsp. *funduliforme* isolates, but none are available for any bovine *F. necrophorum* isolate.

*F. necrophorum* subsp. *funduliforme* B35 was isolated in 1986 from an abscess in the liver of a feedlot steer from a Kansas abattoir (5). This isolate has been characterized with respect to leukotoxin biology (6), outer membrane protein profile (7), and adherence (8) and so was selected for sequence determination. Genomic DNA was subjected to 454 sequencing at The Genome Institute, Washington University, MO. Newbler assembly (Roche) of the 336,159 reads yielded 40 contigs (*N*_50_ size, 161,550 bp; largest contig, 324,876 bp) with 59× coverage. The draft sequence was annotated using the PGAP pipeline (NCBI), resulting in the identification of 1,990 genes, including 1,907 open reading frames (ORFs), 48 tRNAs, and 20 rRNAs. Mutations in 13 pseudogenes were verified by PCR and Sanger sequencing. The G+C content is 34.98%.

The genome size of 2,088,497 kb is within the range (1.96 to 2.31 Mb) determined for four human *F. necrophorum* subsp. *funduliforme* isolates. Among the unique features of the *F. necrophorum* subsp. *funduliforme* B35 genome is the presence of an operon encoding a choline transporter, choline kinase, cholinephosphotransferase, and choline-phosphate cytidylyltransferase, which is linked to lipopolysaccharide (LPS) biosynthesis genes. These features suggest that *F. necrophorum* subsp. *funduliforme* B35 modulates the bovine host response through LPS decoration with phosphocholine (9). These genes are not present in the four human *F. necrophorum* subsp. *funduliforme* genomes. Additional surface-related genes include those encoding an adhesin-filamentous hemagglutinin (FhaB) protein that is present in only one of the 4 sequenced human strains and multiple large exoproteins of the autotransporter and hemagglutinin families.

Four distinct clustered regularly interspaced short palindromic repeat (CRISPR) loci are present, one of which is also present in one of the four human isolates. Each locus harbors a different direct repeat, and a total of 165 CRISPR spacers are present. Despite such potential barriers to horizontal gene transfer, a novel integrative conjugative unit with similarity to *Streptococcus pyogenes* ICE*Sp*1108 (10) and related elements is downstream of *rumA*.

The *F. necrophorum* subsp. *funduliforme* B35 data set presented herein is the first sequence for a bovine *F. necrophorum* isolate. This will allow comparisons to bovine *F. necrophorum* subsp. *necrophorum* strains associated with foot rot or liver abscesses in addition to *F. necrophorum* subsp. *funduliforme* isolates from human patients with Lemierre’s syndrome.

### Nucleotide sequence accession number.

This whole-genome shotgun project has been deposited at DDBJ/EMBL/GenBank under the accession no. AOJP00000000. The version described in this paper is the first version.

## References

[1] Tadepalli S Narayanan SK Stewart GC Chengappa MM Nagaraja TG 2009 *Fusobacterium necrophorum*: a ruminal bacterium that invades liver to cause abscesses in cattle. Anaerobe 15:36–43. 10.1016/j.anaerobe.2008.05.005 18595747

[2] Nagaraja TG Narayanan SK Stewart GC Chengappa MM 2005 *Fusobacterium necrophorum* infections in animals: pathogenesis and pathogenic mechanisms. Anaerobe 11:239–246. 10.1016/j.anaerobe.2005.01.007 16701574

[3] Tadepalli S Stewart GC Nagaraja TG Narayanan SK 2008 Human *Fusobacterium necrophorum* strains have a leukotoxin gene and exhibit leukotoxic activity. J. Med. Microbiol. 57:225–231. 10.1099/jmm.0.47598-0 18201990

[4] Kuppalli K Livorsi D Talati NJ Osborn M 2012 Lemierre’s syndrome due to *Fusobacterium necrophorum*. Lancet Infect. Dis. 12:808–815. 10.1016/S1473-3099(12)70089-0 22633566

[5] Lechtenberg KF Nagaraja TG Leipold HW Chengappa MM 1988 Bacteriologic and histologic studies of hepatic abscesses in cattle. Am. J. Vet. Res. 49:58–62 3354968

[6] Tadepalli S Stewart GC Nagaraja TG Narayanan SK 2008 Leukotoxin operon and differential expressions of the leukotoxin gene in bovine *Fusobacterium necrophorum* subspecies. Anaerobe 14:13–18. 10.1016/j.anaerobe.2007.09.001 17988899

[7] Kumar A Peterson G Nagaraja TG Narayanan S 26 5 2013 Outer membrane proteins of *Fusobacterium necrophorum* subsp. *necrophorum* and subsp. *funduliforme*. J. Basic Microbiol. 10.1002/jobm.201200748 23712857

[8] Kumar A Gart E Nagaraja TG Narayanan S 2013 Adhesion of *Fusobacterium necrophorum* to bovine endothelial cells is mediated by outer membrane proteins. Vet. Microbiol. 162:813–818. 10.1016/j.vetmic.2012.10.022 23153522

[9] Clark SE Weiser JN 2013 Microbial modulation of host immunity with the small molecule phosphorylcholine. Infect. Immun. 81:392–401. 10.1128/IAI.01168-12 23230294 PMC3553803

[10] Brenciani A Tiberi E Bacciaglia A Petrelli D Varaldo PE Giovanetti E 2011 Two distinct genetic elements are responsible for *erm*(TR)-mediated erythromycin resistance in tetracycline-susceptible and tetracycline-resistant strains of *Streptococcus pyogenes*. Antimicrob. Agents Chemother. 55:2106–2112. 10.1128/AAC.01378-10 21343455 PMC3088260

